# Sperm morphology, adenosine triphosphate (ATP) concentration and swimming velocity: unexpected relationships in a passerine bird

**DOI:** 10.1098/rspb.2016.1558

**Published:** 2016-08-31

**Authors:** Clair Bennison, Nicola Hemmings, Lola Brookes, Jon Slate, Tim Birkhead

**Affiliations:** 1 Department of Animal and Plant Sciences, The University of Sheffield, Alfred Denny Building, Western Bank, Sheffield, UK; 2 Zoological Society of London, London, UK

**Keywords:** passerine sperm, sperm competition, sperm energetics, sperm motility, zebra finch

## Abstract

The relationship between sperm energetics and sperm function is poorly known, but is central to our understanding of the evolution of sperm traits. The aim of this study was to examine how sperm morphology and ATP content affect sperm swimming velocity in the zebra finch *Taeniopygia guttata*. We exploited the high inter-male variation in this species and created extra experimental power by increasing the number of individuals with very long or short sperm through artificial selection. We found a pronounced quadratic relationship between total sperm length and swimming velocity, with velocity increasing with length up to a point, but declining in the very longest sperm. We also found an unexpected negative association between midpiece length and ATP content: sperm with a short midpiece generally contained the highest concentration of ATP. Low intracellular ATP is therefore unlikely to explain reduced swimming velocity among the very longest sperm (which tend to have a shorter midpiece).

## 1. Introduction

Sperm morphology evolves rapidly in internally fertilizing species in response to selection inside the female reproductive tract, and this has resulted in dramatic diversification across species [1]. Despite the potential importance of this variation for the formation and maintenance of species [2], the relationship between sperm morphology, function and energetics remains poorly understood.

Sperm of internal fertilizers face myriad challenges inside the female reproductive tract, mostly in the context of post-copulatory sexual selection [3]. In birds, for example, sperm must be motile to rapidly traverse the hostile environment of the vagina to gain access to sperm storage sites [4]. Energetically costly traits, such as high swimming velocity and longevity, are therefore likely to be crucial for success [5].

Across species, sperm swimming velocity appears to be strongly influenced by sperm morphology, i.e. the size and shape of sperm components: the head, midpiece and flagellum. Sperm with long flagella, and consequently, a long total length tend to have relatively high swimming velocity (e.g. [6,7]), presumably because the increase in forward propulsion more easily overcomes drag from the head [8]. The relationship between midpiece length and swimming velocity, however, is more complex and differs between species (e.g. [9,10]).

The midpiece contains mitochondria, which produce chemical energy in the form of adenosine triphosphate (ATP) that can be synthesized either via oxidative phosphorylation (OXPHOS) or glycolysis. Although the relative contribution of energy for motility from these two pathways differs between species (see [11]), proper functioning of the mitochondria appears to be consistently vital for normal sperm motility. Reduced motility in human sperm, for example, has been linked to defective mitochondrial structure [12].

A larger midpiece may contain a greater volume of mitochondria [13], therefore generating more energy than a smaller midpiece. However, the evidence is so far equivocal. In Atlantic salmon *Salmo salar* [14], greater concentrations of ATP were recovered from sperm with longer midpieces. Although this study did not quantify swimming velocity, the authors suggested that extra ATP might enhance sperm motility. This idea was supported by a study of nine rodent species [15], which found a positive relationship between ATP concentration and sperm velocity, suggesting that the extra ATP stored by a sperm may result in faster swimming sperm. By contrast, an interspecific comparison of 23 passerine birds [16] found that species with a longer midpiece contained more intracellular ATP, but these energy reserves were unrelated to swimming velocity. These varied findings probably reflect the different selective pressures experienced across taxa, which could mask more subtle functional patterns.

The zebra finch *Taeniopygia guttata* exhibits considerable between-male variation in sperm morphology, including total sperm length (approximate range: 50–80 µm) [17,18]. Crucially, while the dimensions of all sperm components (head, containing the nucleus; midpiece, comprising a single fused mitochondrion wound helically around the flagellum; tail, which is the remainder of the flagellum that is free from the midpiece [19]) vary widely across males, probably due to limited post-copulatory sexual selection [20], they are highly consistent within and between the ejaculates of individual males [21] and highly heritable [17,22].

**Figure 1.**
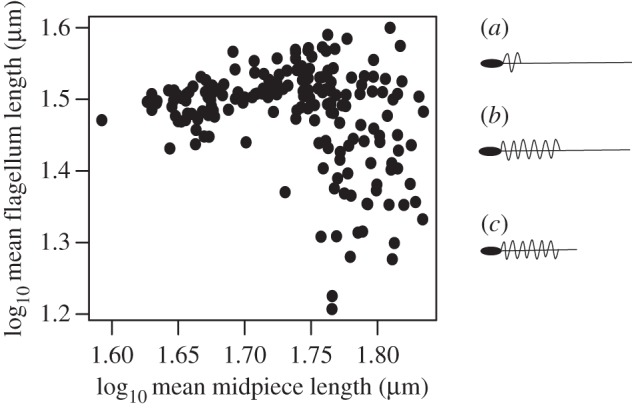
The relationship between midpiece length and flagellum length of sperm (log_10_ of both traits). Each data point is the mean score for a single male. Examples of sperm design (relative lengths of head, midpiece, tail and flagellum) at three areas of the sperm length spectrum are shown (*a*) short midpiece and long flagellum (corresponding with data points top left of plot), (*b*) midpiece and flagellum of approximately equal length (middle right of plot), and (*c*) long midpiece and short flagellum (bottom right of plot).

The morphological ‘design’ of zebra finch sperm (i.e. the relative lengths of the different components) has been explored previously [17,22]. Midpiece and tail length broadly depend on overall flagellum length: midpiece length generally increases with flagellum length, except in the longest sperm which exhibit a spectrum of midpiece lengths from very long to very short (figure 1), with the shortest midpieces being coupled with the longest tails. Previous research on this population of zebra finches [17] found a negative genetic correlation between midpiece and tail length in the longest sperm, resulting in variation in sperm morphology across males. The same phenotypic pattern also exists in wild zebra finches [18] and is underpinned by a genetic effect: gene(s) coding for long flagella also produce short midpieces [17].

Longer zebra finch sperm (with longer flagella) also swim faster [22,23]. In the zebra finch, genes promoting longer flagella—and therefore shorter midpieces—confer faster swimming velocities [22], so the length of the midpiece *per se* may not be the most important factor influencing energetic propulsion. The midpiece structure (e.g. the degree of midpiece coiling around the flagellum, producing the distinctive corkscrew-like motion of passerine sperm [24]) or the amount of stored ATP may have a greater influence on swimming speed than length alone. Therefore, the aim of this study was to quantify the stored energy content within the midpiece, determine the link between sperm morphology and ATP content in the zebra finch, and assess the consequences for sperm swimming velocity.

## 2. Material and methods

Zebra finches were from a captive population at the University of Sheffield and were part of a selective breeding regime to increase the number of males producing either short (less than 60 µm) or long (more than 70 µm) sperm [23], without increasing the range of sperm lengths beyond that occurring naturally [18]. Across males in this study, mean total sperm length ranged from 49.57 to 79.76 µm (mean ± s.d. = 66.38 ± 7.23 µm; electronic supplementary material, table S1). Increasing the number of males producing sperm at either end of the natural length spectrum provided greater experimental power to assess the effects of extreme sperm morphologies. Males were housed in single sex colonies (10 males per 1.2 × 0.5 × 0.4 m cage) with visual and auditory contact with 10 females (housed as above) behind a wire divider. Data were collected across two experimental periods. In 2012–2013, sperm morphology and swimming velocity data were collected from 144 males from three generations (1st = 23 males, 2nd = 25 males, 3rd = 96 males) during a number of smaller studies (data from 42 males were included in [24]). In 2015, morphology and swimming velocity data were collected from an additional 38 males from five generations (1st = 4 males, 2nd = 10 males, 3rd = 14 males, 4th = 8 males, 5th = 2 males) using the same protocol as in 2012–2013, and ATP concentrations were also obtained. Sperm morphology and swimming velocity data were collected by the same observer (C.B.) in all years.

### (a) Sperm collection

Male birds were humanely euthanized in accordance with Schedule 1. Live sperm were collected within 20 min from the left seminal glomerus (SG) and analysed using the Sperm Class Analyzer^®^ (Microptic, Barcelona, Spain), following methods described in [23] (electronic supplementary material).

For the 2015 cohort, the remaining mature sperm (from the distal third portion of the SG) was squeezed into 110 µl of phosphate-buffered saline (PBS) at room temperature (20°C) to avoid activating motility. The sperm suspension was thoroughly aspirated in an eppendorf tube using a pipette and 10 µl was fixed in 90 µl of 5% formalin for sperm concentration and morphology analyses at a later date (see below). The remaining 100 µl was used to quantify ATP content using an ATPlite 300 assay kit (Perkin Elmer, UK) with a modified protocol (described below) allowing sample collection and analysis to be carried out on separate days. ATP was isolated from the sperm suspension by adding 250 µl of PBS, 175 µl of mammalian cell lysis reagent (from ATPlite kit) and incubating at room temperature for 5 min, while mixing with a vortex for 10 s every minute. Samples were centrifuged at 12 000 × *g* for 2.5 min, and the supernatant was snap-frozen in liquid nitrogen and stored at −80°C until quantification.

### (b) Sperm analyses

Sperm swimming velocity, morphology and concentration were analysed as described in [23] (see also the electronic supplementary material).

The ATP content of sperm samples was quantified using a FLUOstar Optima microplate reader (BMG Labtech), which detects bioluminescent output of each sample (electronic supplementary material). Samples were run in triplicate (within-male sample repeatability: *r* = 0.76, *F* = 7.411; *p* < 0.0001 [25]). The blank-corrected mean bioluminescence value per male (mean sample value minus the mean value of all blank wells) was calculated. The concentration of ATP (nmol) per male was calculated by comparison with the standard curve and standardized per 10^6^ sperm, referred to here as stdATP concentration.

### (c) Data analysis

All data were analysed using the base package R v. 2.15.1 [26], unless stated otherwise. The package ‘chemCal’ [27] was used to convert bioluminescence values into ATP concentrations.

As VAP, VCL and VSL were colinear, a principle components analysis (using the function ‘prcomp’) was used to obtain an index of swimming velocity for each individual sperm, which was then used to calculate two average indices of sperm swimming velocity (PC1) for each male: (i) the total sperm mean PC1 (mean value for all sperm in a given sample) and (ii) the fastest 10% mean PC1 (mean value for the fastest 10% of motile sperm in a given sample) [23]. Calculating the PC1 value for the fastest 10% of sperm ensured that the variation in swimming velocity of sperm within each male's sample did not mask associations between PC1 and either morphology or ATP content. Owing to the large variation in swimming velocity in each male's sperm sample, the association between stdATP concentration and swimming velocity was tested using the fastest 10% of sperm only, as this subsample of sperm is likely to include those that would successfully traverse the lower reproductive tract following insemination [22].

The relationships between the different sperm components and PC1 were investigated using individual linear models (using the function ‘lm’), with mean PC1 as the response variable and the mean value per male for each specific sperm component included as the explanatory variable. Relationships between the ratios of the flagellum and head length (flagellum: head) and midpiece and tail length (midpiece: tail) were also examined using the same model structure. Statistical significance was determined against a conservative Bonferroni-corrected critical *p*-value of 0.05/10 (the number of individual comparisons) i.e. 0.005. Weighting the models using the number of sperm contributing to each males PC1 score produced qualitatively similar results (data not shown). The stdATP concentration was log-transformed, and used to examine the relationship between stdATP concentration and both midpiece length and PC1 was examined as above using linear models as above.

## 3. Results

Sperm component lengths varied markedly across males (electronic supplementary material, table S1). Overall, both head and tail length increased with total sperm length (Pearson's correlations, all d.f. = 180; head, *t* = 6.9, *r*^2^ = 0.46, *p* < 0.0001; tail, *t* = 25.4, *r*^2^ = 0.88, *p* < 0.0001), however, the longest sperm tended to have shorter midpieces (Pearson's correlation, d.f. = 180, *t* = −3.9, *r*^2^ = −0.28, *p* < 0.0001), and therefore longer tails. Note that correlations between sperm component lengths and total length are not ideal, because sperm components make up part of the total length—these analyses are included for information only.

### (a) Sperm morphology and swimming velocity

Across all males, there was extensive variation in absolute sperm swimming velocity (electronic supplementary material, table S2). Our analyses revealed a significant positive association between sperm swimming velocity and both head and tail—but not midpiece length—across the entire sperm sample and also in the fastest 10% (electronic supplementary material, table S3). However, because both head and tail length are strongly correlated with total sperm length, these relationships could be driven by an overall positive association between total sperm length and swimming velocity. This is particularly true of head length, which has a negative association with swimming velocity when considered relative to flagellum length (see below).

**Figure 2.**
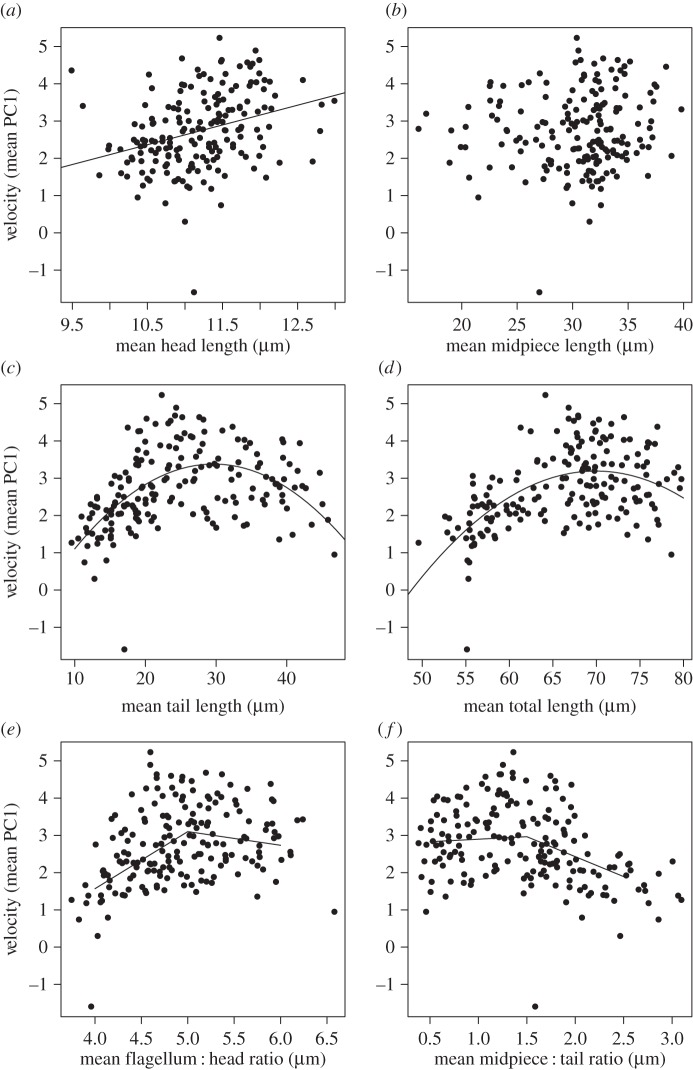
The relationship between swimming velocity (PC1) and (*a*) head, (*b*) midpiece, (*c*) tail, (*d*) total, (*e*) flagellum : head, and (*f*) midpiece : tail of the fastest 10% of sperm per male (*n* = 183). Only midpiece length was unrelated to swimming velocity (PC1). Each data point is the mean score of sperm morphology for a single male. The solid black line shows the predicted relationship from the linear models. See the electronic supplementary material for the model output.

Total sperm length consistently explained the greatest proportion of variation in swimming velocity (although approx. 70% of variation remained unexplained). The relationship between swimming velocity and both total sperm and tail length was quadratic (figure 2*c,d*; electronic supplementary material, table S3), with longer sperm swimming faster up to a point, but with the sperm at the extreme end of the length spectrum showing a decline in swimming speed. While this differs from the positive linear relationship reported in [22], this could be due to the fact that in this study we had greater experimental power to examine the effect of extreme sperm length on swimming velocity, because this study included a greater proportion of birds with very long sperm.

Quadratic relationships also existed between velocity and (i) flagellum : head ratio, and (ii) midpiece : tail ratio (figure 2*e,f*, respectively; electronic supplementary material, table S3). Sperm with long heads relative to the flagellum (low ratios) swam more slowly than sperm of the same length but with smaller heads (high ratios), presumably because a larger head generates more drag [8]. Sperm also swam more slowly when the midpiece was approximately 50% longer than the tail (ratio = 1.5).

### (b) Sperm morphology, adenosine triphosphate concentration and swimming velocity

**Figure 3.**
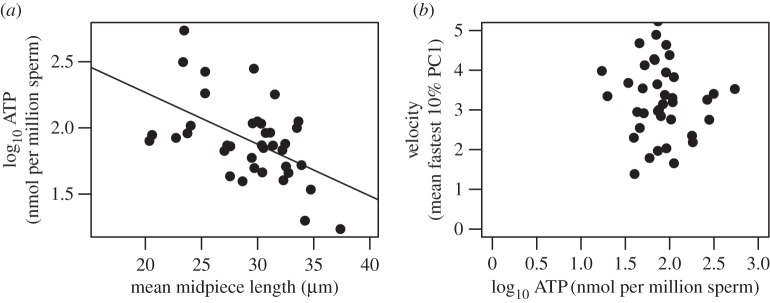
The relationship between intracellular ATP content (log_10_ ATP (nmol per million sperm)) and (*a*) sperm midpiece length; (*b*) sperm swimming velocity (mean fastest 10% PC1). Sperm with a shorter midpiece contain a lower concentration of ATP, but ATP content does not influence swimming velocity.

Contrary to expectation, midpiece length and stdATP concentration were negatively associated, such that the sperm with shorter midpieces contained the highest concentration of ATP (LM, estimate = −0.084 ± 0.025, *t* = −3.29, d.f. = 36, *p* = 0.002; figure 3*a*). Midpiece width was difficult to measure accurately and as a result, repeatability was low (data not included). We were therefore unable to determine whether relative volume of the midpiece varies with length. However, midpiece width is relatively tiny (approx. 3 µm [17]) compared with its length, and little variation was apparent across or between individuals.

Surprisingly, sperm swimming velocity was not influenced either by absolute stdATP concentration (LM, estimate = −0.055 ± 0.127, *t* = −0.436, d.f. = 36, *p* = 0.665; figure 3*b*), or stdATP concentration per unit of midpiece length (LM, estimate = −0.067 ± 0.141, *t* = −0.479, d.f. = 36, *p* = 0.635).

## 4. Discussion

Our results confirm that zebra finch sperm length has a significant impact on sperm swimming velocity. The highest swimming velocity occurred among relatively, but not extremely, long sperm, challenging the widespread view that longer sperm are always faster.

We also found sperm ‘design’—i.e. the relative lengths of sperm components—to be important for swimming velocity. The fastest sperm possess (i) the smallest heads relative to the flagellum and (ii) midpiece and tails of relatively similar lengths. The fact that sperm with shorter midpieces contained higher concentrations of stored intracellular ATP, and this ATP content was unrelated to swimming velocity, suggests that the relationship between energy production and sperm motility is more complex than anticipated.

The effect of sperm length on swimming velocity has been studied in detail across taxa (e.g. [28]). In mice (*Mus domesticus*) [10], sperm with a longer midpiece swim relatively fast, whereas the long sperm of the fruit fly (*Drosophila melanogaster*) [29] swim relatively slowly. In Atlantic salmon (*Salmo salar*) [30], swimming speed is unaffected by sperm morphology. Studies of passerine birds have repeatedly shown that long sperm swim fastest *in vitro* [7,22,31]. Our results are broadly consistent with this, except the very longest zebra finch sperm (which tend to have relatively short midpieces and long tails) swim more slowly.

We recently showed that, in a competitive scenario, males with long sperm fertilize more ova than males with short sperm [23], and attributed this result to the higher swimming velocity of long sperm. However, the results we present here indicate that males producing fairly long, but not the longest, sperm have the greatest potential for achieving fertilization due to their relatively high swimming speeds. It should be noted, however, that recreating the complexity of sperm–female interactions *in vitro* is difficult, and this poses a problem for comparing *in vivo* and *in vitro* results*.* For example, a relatively short snap-shot of swimming velocity *in vitro* does not take into account how fast a sperm swims over the entire period after insemination, or the influence of the reproductive environment.

We initially thought that the decline in velocity observed in the longest sperm might be explained by the relatively small midpiece failing to provide adequate energy to power the extremely long tail. However, sperm with the shortest midpieces actually contained the highest concentration of ATP. This counterintuitive result may be explained by differences in the functioning and/or the packaging of the mitochondria within the midpiece, but we were unable to assess this in this study.

One final aspect of sperm design and its relationship to swimming velocity that we examined was the ratio between the midpiece and tail length. The fastest swimming sperm had midpieces and tails of similar dimensions (as recently reported in the same species at the intra-ejaculate level [32]), suggesting that this particular sperm design may be ‘optimal’. It is possible that the midpiece provides structural support to the sperm [7], maintaining a degree of rigidity to counter the undulation of the tail. If midpiece length falls above or below the ‘optimum’ length relative to the tail, this may reduce swimming efficiency and cause velocity to decline. The ‘optimal’ length could also be influenced by the degree of spiralling of the midpiece around the flagellum, particularly if this influences how sperm swim. This will be an interesting hypothesis to test in the future.

The results of our study, and others (e.g. [16]), raise the important question of why stored ATP content (and mitochondrial haplotype [33]) does not seem to be associated with swimming velocity in passerine sperm, despite the fact that sperm require a source of energy to swim. We propose three possible explanations: (i) energy for swimming could also be produced via another metabolic pathway e.g. glycolysis occurring in the fibrous flagellum sheath. However, in domestic fowl *Gallus domesticus*, glycolysis does not appear to contribute significantly to the sperm's energy budget [34]; whether this is also true for passerines is unknown. (ii) Stored energy may not necessarily be used to achieve faster velocities, but rather to sustain motility or survival for a prolonged period of time, as in [35], which may be beneficial when insemination and fertilization are temporally separated. (iii) Moreover, sperm may not use *all* their stored energy for motility—there are other important processes for which sperm require energy. For example, sperm may need energy to remain viable during storage in the female reproductive tract prior to fertilization [36,37]. It is unknown whether sperm are quiescent inside the sperm storage tubules [38], or if they remain motile [39], but these two scenarios will presumably have different energy requirements. Measuring sperm energy reserves during and post-storage is therefore an important future direction for the study of avian sperm energetics.

## Supplementary Material

ESM1

## Supplementary Material

ESM2

## Supplementary Material

ESM3 - Table S3

## Data accessibility

Datasets are available via Dryad doi:10.5061/dryad.df86m.

## References

[1] Pitnick S, Hosken DJ, Birkhead TR 2009 Sperm morphological diversity. In Sperm biology: an evolutionary perspective (eds Birkhead TR, Hosken DJ, Pitnick S), pp. 69–149. Amsterdam, The Netherlands: Elsevier Ltd.

[2] Howard DJ, Palumi SR, Birge LM, Manier MK 2009 Sperm and speciation. In Sperm biology: an evolutionary perspective (eds Birkhead TR, Hosken DJ, Pitnick S), pp. 367–403. Amsterdam, The Netherlands: Elsevier Ltd.

[3] Birkhead TR, Møller AP, Sutherland WJ 1993 Why do females make it so difficult for males to fertilize their eggs? J. Theor. Biol. 161, 51–60. (10.1006/jtbi.1993.1039)

[4] Bakst MR, Wishart G, Brillard JP 1994 Oviducal sperm selection, transport, and storage in poultry. Poult. Sci. Rev. 5, 117–143.

[5] Donoghue AM, Sonstegard TS, King LM, Smith EJ, Burt DW 1999 Turkey sperm mobility influences paternity in the context of competitive fertilization. Biol. Reprod. 61, 422–427. (10.1095/biolreprod61.2.422) 10411522

[6] Gomendio M, Roldan ERS 2008 Implications of diversity in sperm size and function for sperm competition and fertility. Int. J. Dev. Biol. 52, 439–447. (10.1387/ijdb.082595mg) 18649256

[7] Lüpold S, Calhim S, Immler S, Birkhead TR 2009 Sperm morphology and sperm velocity in passerine birds. Proc. R. Soc. B 276, 1175–1181. (10.1098/rspb.2008.1645) PMC2679085 19129098

[8] Humphries S, Evans JP, Simmons LW 2008 Sperm competition: linking form to function. BMC Evol. Biol. 8, 319 (10.1186/1471-2148-8-319) 19032741 PMC2632676

[9] Malo AF, Gomendio M, Garde J, Lang-Lenton B, Soler AJ, Roldan ERS 2006 Sperm design and sperm function. Biol. Lett. 2, 246–249. (10.1098/rsbl.2006.0449) 17148374 PMC1618917

[10] Firman RC, Simmons LW 2010 Sperm midpiece length predicts sperm swimming velocity in house mice. Biol. Lett. 6, 513–516. (10.1098/rsbl.2009.1027) 20147311 PMC2936205

[11] Turner RM 2003 Tales from the tail: what do we really know about sperm motility? J. Androl. 24, 790–803. (10.1002/j.1939-4640.2003.tb03123.x) 14581499

[12] Mundy AJ, Ryder TA, Edmunds DK 1995 Asthenozoospermia and the human sperm mid-piece. Hum. Reprod. 10, 116–119. (10.1093/humrep/10.1.116) 7745038

[13] Anderson MJ, Dixson AF 2002 Sperm competition—motility and the midpiece in primates. Nature 416, 496 (10.1038/416496a) 11932733

[14] Vladić TV, Afzelius BA, Bronnikov GE 2002 Sperm quality as reflected through morphology in salmon alternative life histories. Biol. Reprod. 66, 98–105. (10.1095/biolreprod66.1.98) 11751270

[15] Tourmente M, Rowe M, Mar Gonzalez-Barroso M, Rial E, Gomendio M, Roldan ERS 2013 Postcopulatory sexual selection increases ATP content in rodent spermatozoa. Evolution 67, 1838–1846. (10.1111/evo.12079) 23730775

[16] Rowe M, Laskemoen T, Johnsen A, Lifjeld JT 2013 Evolution of sperm structure and energetics in passerine birds. Proc. R. Soc. B 280, 20122616 (10.1098/rspb.2012.2616) PMC3574354 23282997

[17] Birkhead TR, Pellatt EJ, Brekke P, Yeates R, Castillo-Juarez H 2005 Genetic effects on sperm design in the zebra finch. Nature 434, 383–387. (10.1038/nature03374) 15772662

[18] Immler S, Griffith SC, Zann R, Birkhead TR 2012 Intra-specific variance in sperm morphometry: a comparison between wild and domesticated zebra finches *Taeniopygia guttata*. Ibis 154, 480–487. (10.1111/j.1474-919X.2012.01232.x)

[19] Jamieson BGM 2007 Avian spermatozoa: structure and phylogeny. In Reproductive biology and phylogeny of birds, *vol. 6A* (ed. Jamieson BGM), pp. 349–511. Enfield, UK: Science Publishers.

[20] Calhim S, Immler S, Birkhead TR 2007 Postcopulatory sexual selection is associated with reduced variation in sperm morphology. PLoS ONE 2, e413 (10.1371/journal.pone.0000413) 17476335 PMC1855076

[21] Birkhead TR, Fletcher F 1995 Male phenotype and ejaculate quality in the zebra finch *Taeniopygia guttata*. Proc. R. Soc. Lond. B 262, 329–334. (10.1098/rspb.1995.0213) 8587890

[22] Mossman J, Slate J, Humphries S, Birkhead T 2009 Sperm morphology and velocity are genetically co-determined in the zebra finch. Evolution 63, 2730–2737. (10.1111/j.1558-5646.2009.00753.x) 19552737

[23] Bennison C, Hemmings N, Slate J, Birkhead T 2015 Long sperm fertilize more eggs in a bird. Proc. R. Soc. B 282, 20141897 (10.1098/rspb.2014.1897) PMC4286041 25621327

[24] Vernon GG, Woolley DM 1999 Three-dimensional motion of avian spermatozoa. Cell Motil. Cytoskel. 42, 149–161. (10.1002/(sici)1097-0169(1999)42:2%3C149::aid-cm6%3E3.0.co;2-0) 10215424

[25] Lessells CM, Boag PT 1987 Unrepeatable repeatabilities—a common mistake. Auk 104, 116–121. (10.2307/4087240)

[26] R Development Core Team. 2012 R: a language and environment for statistical computing. Vienna, Austria: R Foundation for Statistical Computing See http://www.R-project.org/.

[27] Ranke J 2013 chemCal: calibration functions for analytical chemistry. R package version 0.1-29. http://CRAN.R-project.org/package=chemCal.

[28] Simpson JL, Humphries S, Evans JP, Simmons LW, Fitzpatrick JL 2014 Relationships between sperm length and speed differ among three internally and three externally fertilizing species. Evolution 68, 92–104. (10.1111/evo.12199) 24224469

[29] Lüpold S, Manier MK, Berben KS, Smith KJ, Daley BD, Buckley SH, Belote JM, Pitnick S 2012 How multivariate ejaculate traits determine competitive fertilization success in *Drosophila melanogaster*. Curr. Biol. 22, 1667–1672. (10.1016/j.cub.2012.06.059) 22840512

[30] Gage MJG, MacFarlane C, Yeates S, Shackleton R, Parker GA 2002 Relationships between sperm morphometry and sperm motility in the Atlantic salmon. J. Fish Biol. 61, 1528–1539. (10.1006/jfbi.2002.2171)

[31] Helfenstein F, Podevin M, Richner H 2010 Sperm morphology, swimming velocity, and longevity in the house sparrow *Passer domesticus*. Behav. Ecol. Sociobiol. 64, 557–565. (10.1007/s00265-009-0871-x)

[32] Hemmings N, Bennison C, Birkhead TR 2016 Intra-ejaculate sperm selection in female zebra finches. Biol. Lett. 12, 20160220 (10.1098/rsbl.2016.0220) 27277953 PMC4938051

[33] Mossman JA, Slate J, Birkhead TR 2010 Mitochondrial haplotype does not affect sperm velocity in the zebra finch *Taeniopygia guttata*. J. Evol. Biol. 23, 422–432. (10.1111/j.1420-9101.2009.01913.x) 20040001

[34] Froman DP, Feltmann AJ 1998 Sperm mobility: a quantitative trait of the domestic fowl (*Gallus domesticus*). Biol. Reprod. 58, 379–384. (10.1095/biolreprod58.2.379) 9475392

[35] Dziminski MA, Roberts JD, Beveridge M, Simmons LW 2009 Sperm competitiveness in frogs: slow and steady wins the race. Proc. R. Soc. B 276, 3955–3961. (10.1098/rspb.2009.1334) PMC2825793 19710059

[36] Bobr LW, Ogasawara FX, Lorenz FW 1964 Distribution of spermatozoa in the oviduct and fertility in domestic birds. 1. Residence sites of spermatozoa in fowl oviducts. J. Reprod. Fertil. 8, 39–47. (10.1530/jrf.0.0080039) 14195706

[37] Briskie JV, Montgomerie R 1993 Patterns of sperm storage in relation to sperm competition in passerine birds. Condor 95, 442–454. (10.2307/1369366)

[38] Bakst MR 1985 Zinc reduces turkey sperm oxygen uptake *in vitro*. Poult. Sci. 64, 564–566. (10.3382/ps.0640564) 3991430

[39] Froman D 2003 Deduction of a model for sperm storage in the oviduct of the domestic fowl (*Gallus domesticus*). Biol. Reprod. 69, 248–253. (10.1095/biolreprod.102.013482) 12646493

