# Supplementary material for: Sperm morphology, adenosine triphosphate (ATP) concentration and swimming velocity: unexpected relationships in a passerine bird
Source: Proc Biol Sci. 2016 Aug 31;283(1837):20161558. doi: 10.1098/rspb.2016.1558 (PMC5013805; doi:10.1098/rspb.2016.1558)
Supplement: ESM1 [file rspb20161558supp1.docx]

Sperm morphology and swimming velocity summary data

Table S1. Summary data for the sperm morphology from 182 males. The average measurement per male was based on ten measured sperm. Standard deviation (SD) and coefficient of variation (CV) are included.

| Component | Range (µm) | Mean ± SD | CV |
| --- | --- | --- | --- |
| Head | 9.39 – 12.99 | 11.19 ± 0.62 | 5.55 |
| Midpiece | 16.11 – 39.80 | 30.47 ± 4.47 | 14.67 |
| Tail | 9.55 – 46.74 | 24.72 ± 9.28 | 37.55 |
| Flagellum | 39.12 – 68.33 | 55.19 ± 6.97 | 12.63 |
| Total | 49.57 – 79.76 | 66.38 ± 7.23 | 10.90 |

Table S2. Summary data for the sperm swimming velocity from 182 males. The average measurement per male in the total sperm population was based on at least 100 sperm.

| Subpopulation | Range (µm/s) | Mean ± SD | CV |
| --- | --- | --- | --- |
| Total |  |  |  |
| VAP | 0.26 – 87.42 | 42.89 ± 15.76 | 36.75 |
| VCL | 0.75 – 89.83 | 53.53 ± 16.91 | 31.58 |
| VSL | 0.09 – 83.12 | 39.08 ± 15.83 | 40.51 |
| Fastest 10% |  |  |  |
| VAP | 2.59 – 117.12 | 73.18 ± 18.44 | 25.20 |
| VCL | 7.40 – 117.72 | 83.81 ± 15.41 | 18.39 |
| VSL | 0.90 – 114.92 | 69.67 ± 19.06 | 27.36 |
