# Supplementary material for: Sperm morphology, adenosine triphosphate (ATP) concentration and swimming velocity: unexpected relationships in a passerine bird
Source: Proc Biol Sci. 2016 Aug 31;283(1837):20161558. doi: 10.1098/rspb.2016.1558 (PMC5013805; doi:10.1098/rspb.2016.1558)
Supplement: ESM2 [file rspb20161558supp2.docx]

Supplementary materials and methods

Sperm collection

Live sperm were collected within 20 minutes of euthanasia from the left seminal glomerus (SG). Using fine forceps, mature sperm were squeezed from the initial 1 mm (approx.) of the distal end of the SG into a small petri dish containing warm (38^o^C) Ham’s F10 nutrient media (Life Technologies™). The sperm diffuse as they swim out from the SG into the surrounding media, forming a ‘cloud’. After 10 s, 4 µl of sperm solution (approximately 8 x 10^6^ per ml) was collected from the edge of the cloud to analyse swimming velocity.

Sperm velocity

Videos of swimming sperm were captured and analysed using the Sperm Class Analyzer® (Microptic, Barcelona, Spain), in pseudo-negative phase at 200x magnification with a Basler acA780-75gc camera connected to an Olympus BX41 microscope. Four microlitres of sperm solution was loaded into a pre-warmed (38^o^C) 20 µm depth slide chamber (Leja®, Netherlands) on a heated microscope stage. Multiple 1 s video clips (50 frames per second) per male were recorded in a systematic manner until at least 100 sperm had been tracked. Prior to analysis, any debris incorrectly identified as sperm by the software were manually deleted. Three kinematic parameters from each sperm were obtained: (i) average path velocity (VAP), (ii) curvilinear velocity (VCL), and (iii) straight line velocity (VSL). The kinematic values of drifting sperm (obtained by analysing videos of dead sperm) were set to zero (i.e. non-motile) when the following conditions were met: VAP ≤ 7.5 µm/s; VCL ≤ 14 µm/s; VSL ≤ 2.5 µm/s [1].

Sperm morphology

Ten intact, morphologically normal sperm from each male were photographed using light microscopy (Leitz Laborlux S) at 400x and an Infinity 3 camera (Luminera Corporation). The length of sperm components (head, coiled midpiece and tail) was measured to the nearest 0.01 µm using ImageJ [2]. Observer measurement repeatability [3] was high for all sperm components (head: F = 74.9: r = 0.97; midpiece: F = 1390.1: r = 0.99; tail: F = 616.3: r = 0.98). As sperm morphology is repeatable within and between ejaculates of individual males [4], the mean value of each sperm component was calculated for each male.

Sperm concentration

Sperm concentration was estimated using an Improved Neubauer chamber. The mean number of sperm was calculated across two counting chambers, and sperm concentration expressed as 10^6^ sperm per ml.

ATP assay protocol

The ATP content of the sperm samples was quantified using a FLUOstar Optima microplate reader (BMG Labtech), which detects bioluminescent output of each sample. ATP standard solutions were prepared following the kit instructions, run in triplicate (from 1 x 10^-3^ to 1 x 10^-10^ mol) on each microplate, and averaged to give an ATP standard curve (r^2^ = 0.99). Sperm samples were prepared for analysis as follows: each microplate sample well contained 150 µl of sperm supernatant (defrosted at room temperature immediately prior to analysis, containing 100 µl of diluted sperm suspension and 50 µl cell lysis reagent). Fifty microliters of reconstituted substrate solution was added to each microplate well. ‘Blank’ wells - containing all reagents as above except sperm supernatant - were included in each row to give an average background bioluminescence. The plate was dark-adapted for 5 minutes and shaken for an additional 5 minutes in the dark at 300 rpm using orbital shaker inside the plate reader. Finally, bioluminescence was detected using the ‘lens’ setting, in order to detect all bioluminescence output at the full range of wavelengths.

References

1. Bennison et al. 2015. Long sperm fertilize more eggs in a bird. *Proc. R. Soc. Lond. B* **282,** 20141897. (doi:10.1098/rspb.2014.1897).

2. Schneider, CA, Rasband, WS & Eliceiri, KW . 2012. NIH Image to ImageJ: 25 years of image analysis. Nat. Methods 9, 671-675. (doi:10.1038/nmeth.2089).

3. Lessells, CM & Boag, PT. 1987 Unrepeatable repeatabilities - a common mistake. *Auk* **104**, 116-121. (doi:10.2307/4087240).

4. Birkhead, TR & Fletcher, F. 1995 Male phenotype and ejaculate quality in the zebra finch *Taeniopygia guttata*. *Proc. R. Soc. Lond. B* **262**, 329–334. ([doi:10.1098/rspb.1995.0213](http://dx.doi.org/10.1098/rspb.1995.0213))
