# Supplementary material for: Sperm morphology, adenosine triphosphate (ATP) concentration and swimming velocity: unexpected relationships in a passerine bird
Source: Proc Biol Sci. 2016 Aug 31;283(1837):20161558. doi: 10.1098/rspb.2016.1558 (PMC5013805; doi:10.1098/rspb.2016.1558)
Supplement: ESM3 - Table S3 [file rspb20161558supp3.docx]

Table S3. The results of models analysing the effect of six measurements of sperm morphology on swimming velocity (PC1). Head and midpiece length were modeled as linear explanatory variables, whereas all other components were modeled by including a quadratic term^a^. Model estimates for both the linear and quadratic terms are presented. All measures of sperm length, except midpiece length were significantly associated with PC1, in both sperm populations (p < 0.05) and are in bold.

|  |  |  |  |  |  |  |
| --- | --- | --- | --- | --- | --- | --- |
| Sperm population | Sperm component | Estimate ± SD | t | F _1,180_  F _2,179_^a^ | Adjusted R^2^ | p |
|  | Head | 0.53 ± 0.12 | 4.57 | 20.88 | 0.099 | **< 0.0001** |
| 10% | Midpiece | 0.02 ± 0.02 | 1.16 | 1.35 | 0.002 | 0.247 |
|  | Tail  Tail^a^ | 0.35 ± 0.04  -0.006 ± 0.0008 | 8.36  -7.73 | 39.89 ^a^ | 0.300 | **< 0.0001** |
|  | Total length  Total length^a^ | 0.99 ± 0.16  -0.007 ± 0.001 | 6.12  -5.75 | 41.68 ^a^ | 0.310 | **<0.0001** |
|  | Flagellum: head  Flagellum: head^a^ | 10.07 ± 1.67  -0.95 ± 0.17 | 6.04  -5.72 | 28.03 ^a^ | 0.230 | **< 0.0001** |
|  | Midpiece: tail  Midpiece: tail^a^ | 1.34 ± 0.46  -0.60 ± 0.14 | 5.69  -6.15 | 22.22 ^a^ | 0.190 | **<0.0001** |
|  |  |  |  |  |  |  |
|  | Head | 0.46 ± 0.11 | 4.24 | 17.99 | 0.086 | **< 0.0001** |
| Total | Midpiece | 0.02 ± 0.02 | 1.26 | 1.58 | 0.003 | 0.211 |
|  | Tail  Tail^a^ | 0.22 ± 0.043  -0.004 ± 0.0007 | 5.21  -4.88 | 14.82 ^a^ | 0.133 | **<0.0001** |
|  | Total length  Total length^a^ | 0.64 ± 0.17  -0.005 ± 0.001 | 3.83  -361 | 15.83 ^a^ | 0.141 | **<0.0001** |
|  | Flagellum: head  Flagellum: head^a^ | 6.75 ± 1.66  -0.65 ± 0.17 | 4.07  -3.92 | 10.38 ^a^ | 0.094 | **<0.0001** |
|  | Midpiece: tail  Midpiece: tail^a^ | 0.98 ± 0.45  -0.41± 0.14 | 2.18  -2.94 | 8.79 ^a^ | 0.079 | **0.0002** |
